# Logistic Regression Is Non-Inferior to the Response Surface Model in Patient Response Prediction of Video-Assisted Thoracoscopic Surgery

**DOI:** 10.3390/ph17010095

**Published:** 2024-01-10

**Authors:** Hui-Yu Huang, Shih-Pin Lin, Hsin-Yi Wang, Jing-Yang Liou, Wen-Kuei Chang, Chien-Kun Ting

**Affiliations:** 1 Department of Anesthesiology, Taipei Veterans General Hospital and National Yang Ming Chiao Tung University, Taipei 112201, Taiwan; karen93093@gmail.com (H.-Y.H.); aslam.lin@gmail.com (S.-P.L.); vicky8101@gmail.com (H.-Y.W.);; 2 Institute of Emergency and Critical Care Medicine, National Yang Ming Chiao Tung University, Taipei 112304, Taiwan

**Keywords:** anesthesia, logistic regression, response surface model, receiver operating characteristic

## Abstract

Response surface models (RSMs) are a new trend in modern anesthesia. RSMs have demonstrated significant applicability in the field of anesthesia. However, the comparative analysis between RSMs and logistic regression (LR) in different surgeries remains relatively limited in the current literature. We hypothesized that using a total intravenous anesthesia (TIVA) technique with the response surface model (RSM) and logistic regression (LR) would predict the emergence from anesthesia in patients undergoing video-assisted thoracotomy surgery (VATS). This study aimed to prove that LR, like the RSM, can be used to improve patient safety and achieve enhanced recovery after surgery (ERAS). This was a prospective, observational study with data reanalysis. Twenty-nine patients (American Society of Anesthesiologists (ASA) class II and III) who underwent VATS for elective pulmonary or mediastinal surgery under TIVA were enrolled. We monitored the emergence from anesthesia, and the precise time point of regained response (RR) was noted. The influence of varying concentrations was examined and incorporated into both the RSM and LR. The receiver operating characteristic (ROC) curve area for Greco and LR models was 0.979 (confidence interval: 0.987 to 0.990) and 0.989 (confidence interval: 0.989 to 0.990), respectively. The two models had no significant differences in predicting the probability of regaining response. In conclusion, the LR model was effective and can be applied to patients undergoing VATS or other procedures of similar modalities. Furthermore, the RSM is significantly more sophisticated and has an accuracy similar to that of the LR model; however, the LR model is more accessible. Therefore, the LR model is a simpler tool for predicting arousal in patients undergoing VATS under TIVA with Remifentanil and Propofol.

## 1. Introduction

Enhanced recovery after surgery (ERAS) represents a novel trend in the realm of perioperative medicine. It signifies a meticulously crafted, evidence-based perioperative pathway that has been developed by a multidisciplinary team. The overarching goal of ERAS is to reduce the surgical stress experienced by patients, expedite the process of recovery, and reduce the occurrence of postoperative complications [1,2]. Its applicability extends across a variety of surgical procedures [3,4,5,6,7,8], and our specific objective was to realize early extubation within the context of ERAS [9].

Within the domain of thoracic surgery, video-assisted thoracoscopic surgery (VATS) is a widely employed minimally invasive technique. Numerous studies have demonstrated the manifold advantages conferred by ERAS, which include a reduction in the incidence of air leaks and atelectasis, as well as a decreased requirement for bronchoscopic aspiration [10,11]. Consequently, the implementation of ERAS has the potential to curtail the duration of postoperative hospital stays and lower the rate of postoperative complications, demonstrating its significant utility in optimizing patient outcomes.

Understanding pharmacodynamics principles pertaining to the onset and offset profiles of drugs is of paramount importance in the field of anesthetic practice. A novel trend in contemporary anesthesia, the response surface model (RSM), has garnered significant attention [12]. The RSM, at its core, is rooted in the adoption of a mathematical model that encompasses elements such as isobolograms, concentration–effect curves, and curve shift effects. It is noteworthy that the RSM is particularly suited for a two-drug model; however, the graphical analysis of a three-drug (or more) model proves to be a more intricate endeavor. The versatility of the RSM is highlighted by its ability to employ various models that enable the prediction of essential clinical parameters, including patients’ wake-up time, tolerance to stimuli, and other clinical presentations [13,14]. Furthermore, the potential to create multiple combinations using various drug concentrations significantly enhances precision and safety in clinical practice. The clinical applicability of the RSM has been substantiated by a wealth of research studies [15].

On the other hand, logistic regression (LR) emerges as a precise and relatively straightforward method with wide-ranging applications across multiple domains. Widely used in data analysis and prediction, LR is particularly effective when the outcome of interest falls into one of two categories, such as success or failure, yes or no. This modeling approach provides valuable insights into the likelihood of an event occurring based on input variables. LR is extensively utilized in medical research, economics, marketing, and numerous other disciplines, making it a versatile tool for predicting and understanding binary outcomes in diverse contexts. LR is widely available in many statistical software packages, including SPSS Statistics 20, which underscores its accessibility and widespread use. In contrast, the RSM often requires custom coding and is not typically included in most statistical software suites. The appeal of LR partly lies in its simplicity; it offers straightforward interpretation and ease of implementation. Essentially, it involves fitting a linear boundary within a transformed space to differentiate between two classes. Furthermore, LR employs fewer parameters compared to the RSM, enhancing its practicality in various analytical scenarios. Our working hypothesis centers on the feasibility of applying LR within clinical practice to predict patient responses in comparison to the RSM, thus offering a potentially valuable and more accessible alternative.

This study aimed to prove that LR, like the RSM, can improve patient safety and achieve ERAS by comparing performance and receiver operating characteristic (ROC) curves. The primary endpoint of our study was to hypothesize that a simpler model, such as LR, can predict patient responses like the RSM. The secondary endpoint was whether LR could predict a patient’s response with high accuracy.

## 2. Results

### 2.1. Patient Profiles

Twenty-nine patients (American Society of Anesthesiologists (ASA) class II to III) who underwent VATS for elective pulmonary or mediastinal surgery under TIVA were included. This study included 11 males and 18 females. The mean age was approximately 54.6 years, mean body height was 159.5 cm, mean body weight was 60.6 kg, and body mass index was 23.8 kg/m^2^. The average operation time was 198 min.

### 2.2. Pharmacological Profiles

The Remifentanil and Propofol concentrations were in the ranges of 0–5.268 and 0–7.890, respectively. Notably, significant concentration variations are common during the induction or emergence phases of anesthesia.

### 2.3. Response Surface Model

**Figure 1:**
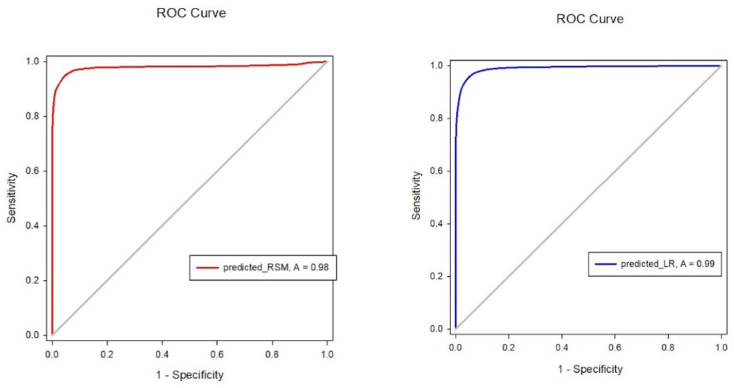
Receiver operating characteristic (ROC) curve area for response surface model (RSM; red line) and logistic regression (LR; blue line). The 95% confidence intervals for the ROC curve area were 0.987–0.990 for RSM and 0.992–0.994 for LR.

The prediction accuracy was 93.2%. The ROC curve analysis shown in the left image of Figure 1 for the RSM indicated an ROC curve area of 0.988 with a 95% confidence interval of 0.987 to 0.990. The area under the ROC curve was generated using SigmaPlot software, version 12.5.

### 2.4. Logistic Regression Model

An SPSS logistic regression model was used. Since we intended to calculate the probability of regaining response, we assumed the time when the patient would be awake was 1 (100% of regaining response) and the time of LOR was 0 (0% of regaining response). The final model was constructed using the concentration sets of Remifentanil and Propofol. Over 57,000 concentration sets were used to construct the model. The coefficient estimate for Remifentanil and Propofol was 2.652 and −3.403, respectively. The prediction accuracy was 95%. ROC curve analysis for the logistic model shown in the right image of Figure 1 indicated an ROC curve area of 0.989, with a 95% confidence interval of 0.989–0.990. The secondary endpoint was whether LR could predict a patient’s response with high accuracy, and with a prediction accuracy of 95%, we believe that logistic regression can predict the emergence of patients undergoing VATS with high accuracy.

### 2.5. Comparison

**Figure 2:**
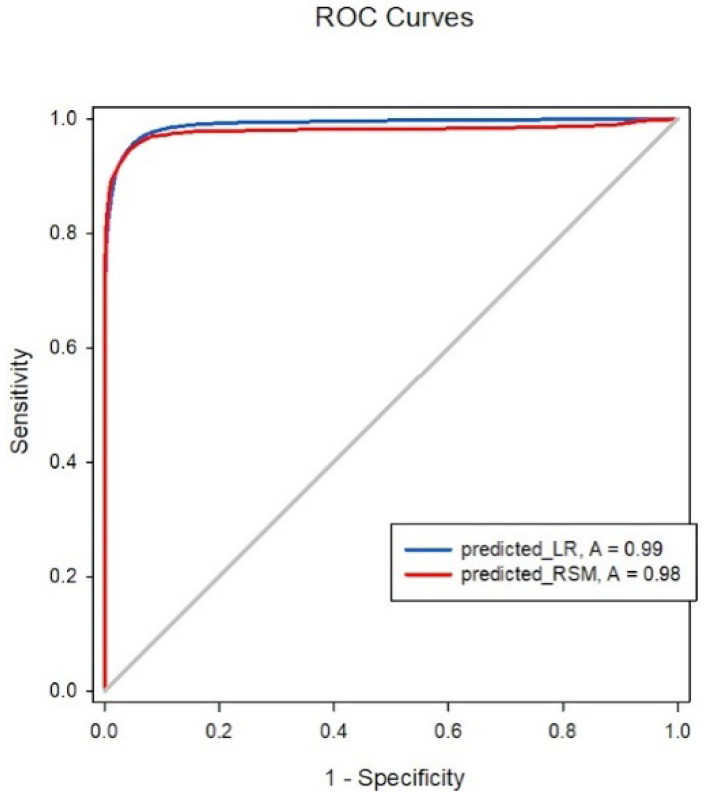
ROC analysis comparison between LR and RSM.

The isobolographic plot depicts the synergism between Remifentanil and Propofol. Based on the previous definition of predictive accuracy, the Greco model was 93.2% accurate; however, the LR model exhibited 95% accuracy using the same standard. Figure 2 demonstrates the ROC curve area for the Greco and LR models was 0.979 and 0.989, respectively. No significant differences were observed between the two models in predicting the probability of regaining response. Regarding our primary endpoint, we hypothesized that a simpler model, such as LR, could predict a patient’s response like the RSM. The results proved our hypothesis; when the model was constructed with the concentration sets of Remifentanil and Propofol, the LR models were non-inferior to the RSMs.

## 3. Discussion

The primary endpoint of our study was to hypothesize that a simpler model, such as LR, can predict patient responses like the RSM. The secondary endpoint was whether LR could predict a patient’s response with high efficiency. Generally, the Greco and LR models accurately predicted the regained response (RR) in VATS.

### 3.1. Logistic Regression

LR is a statistical modeling technique extensively used in medical research and analysis. This model is tailor-made to evaluate the association between multiple predictor variables and a binary outcome variable [16,17]. The coefficients associated with the predictor variables can be determined by fitting the LR model to the observed data, enabling quantification of their respective influences on the outcome. Numerous studies have incorporated demographic and anesthesiological factors into logistic regression (LR) models for forecasting the trio of prevalent complications associated with spinal anesthesia: hypotension, bradycardia, and nausea [18]. This study demonstrated reliable predictive results; therefore, we believe that LR can be applied in the field of anesthesia.

### 3.2. Response Surface Model

The Greco model, integrated within the broader framework of response surface modeling, stands as a significant advancement in pharmacodynamic modeling. At its core, the Greco model operates under the assumption that each drug within a combination can independently exert its specific effect, thereby enabling a thorough investigation of the complex pharmacological interactions that define drug synergy or antagonism. The Greco model’s versatility extends to its application in various therapeutic domains, including anesthesia and sedation, where the precise coordination of multiple drugs is crucial for optimal patient outcomes. By utilizing mathematical formulations, such as isobolograms, concentration–effect curves, and curve shift effects, the Greco model provides a quantitative and predictive framework for assessing drug interactions.

However, a limitation arises when incorporating opioids into a hypnosis model, as it may erroneously suggest that opioids alone can reliably induce hypnosis. The model’s assumption that opioids alone can reliably induce hypnosis may oversimplify the multifaceted nature of opioid pharmacodynamics, as opioids are known for their variable and inconsistent effects on hypnotic states.

### 3.3. Logistic Regression vs. Response Surface Model

The RSM and LR are extensively used statistical approaches in medical research for modeling and analyzing complex relationships between predictor variables and outcomes. The RSM utilizes polynomial functions to construct a fitted surface representing the relationship between predictors and outcomes. However, LR is a simpler and more practicable technique. The simplicity of LR lies in its ease of interpretation, as the estimated coefficients provide direct insights into the direction and magnitude of the predictor effects.

### 3.4. Enhanced Recovery after Surgery

ERAS, a multidisciplinary approach aimed at optimizing patient recovery post-surgery, emphasizes evidence-based protocols. The RSM and LR, on the other hand, provide a statistical framework for analyzing complex interactions between variables. The integration of ERAS, the RSM, and LR leverages the precision of the modeling technique to fine-tune and individualize elements of the ERAS pathway. This integration not only enhances the predictive accuracy of patient responses but also contributes to the continual refinement of ERAS protocols, fostering a patient-centric and data-driven approach to perioperative care. Our results showed that the RSM and LR models could precisely predict the RR time from general anesthesia. Therefore, using the LR model, we can determine the extubation time. We can also reduce unnecessary utilization of anesthetic agents. Other studies have shown that ERAS in thoracic surgery can significantly reduce the readmission rate [19]. Additionally, given the frequent occurrence of VATS procedures, the implementation of ERAS protocols can effectively reduce preoperative waiting periods and enhance the turnover rate of operating rooms. The minimally invasive nature of VATS aligns with ERAS goals by reducing surgical trauma, minimizing postoperative pain, and expediting patient recovery.

### 3.5. Drug Interaction

The intricate interplay between Propofol and Remifentanil, two commonly employed agents in intravenous anesthesia, constitutes a critical aspect of perioperative pharmacodynamics. Propofol, a sedative–hypnotic agent, exerts its effects primarily through the potentiation of gamma-aminobutyric acid (GABA) receptors, resulting in central nervous system depression. Concurrently, Remifentanil, an ultra-short-acting opioid analgesic, targets mu-opioid receptors, mitigating pain perception. Propofol and Remifentanil, when used in combination, have demonstrated a synergistic impact in enhancing anesthesia effectiveness; Propofol reduces Remifentanil dosage requirements during anesthesia to achieve synergistic inhibition of reactions to procedures such as laryngoscopy, intubation, and surgical incitements [17,18,19,20,21,22,23,24]. In addition, Remifentanil synergistically decreases the dosage requirement of Propofol to achieve anesthesia, which is associated with the return to consciousness. The precise modulation of their concentrations and infusion rates is paramount in tailoring anesthesia to individual patient needs, ensuring optimal sedation and analgesia while minimizing the risk of adverse events.

### 3.6. Limitations

The implementation of the response surface model (RSM) methodology relies on a carefully selected cohort of unstimulated volunteers who are free from surgical pain or the need for endotracheal intubation. It is important to note that utilizing unstimulated volunteer response surface analysis for assessing sedation may not provide a truly accurate reflection of sedation levels observed in patients undergoing surgical procedures that entail the use of a double-lumen tube (DLT) and are associated with surgical injury. This holds true even for exposure to the same concentrations of Propofol and Remifentanil.

Furthermore, it is essential to recognize that the clinical context can significantly impact the performance of the model, as it may be influenced by various confounding variables within the clinical environment. Additionally, one must exercise caution due to the potential for drug interactions occurring at critical concentrations, which further underscores the need for precise and vigilant management.

Conversely, the LR model was constructed utilizing proprietary datasets, and internal validation techniques were utilized to execute receiver operating characteristic (ROC) curve analysis. Internal validation, often referred to as model validation, plays an important role in the assessment of a model’s performance within the same dataset from which it was derived. It serves the crucial function of detecting overfitting while providing an estimation of the model’s predictive accuracy within the confines of the dataset. In contrast, external validation assumes a distinct role by scrutinizing the model’s ability to extrapolate and generalize its efficacy to entirely novel and independent datasets.

The assessment of a patient’s alertness and sedation level was carried out using the Observer’s Assessment of Alertness and Sedation Scale (OAA/S). This scale, originally designed to capture a patient’s state in a subtle manner, was transformed into dichotomous data, classifying patients as either awake or not. However, this simplification of the patient’s condition into binary categories raises a critical concern—it may inevitably result in the loss of valuable data, as it fails to account for the nuances and subtleties that can exist within the spectrum of alertness and sedation in clinical practice.

The sample size requirements differ between LR and the RSM; a small number of 20 patients was required to construct an RSM [25]. Using LR, a consistent AUC was attained with about 20 to 50 events for each variable [26]. In their study on the LR model, van der Ploeg et al. observed that a consistent AUC was obtained with the presence of 20–50 events per variable [27]. We are confident that our sample size satisfies the essential criteria for the development of this model. Our patients were mostly females and middle-aged to elderly, with a mean age of 54.6 years.

Looking ahead, we aspire to extend our investigations by employing the RSM and LR to compare the accuracy of anesthesia emergence across a broader spectrum of surgeries. The chosen threshold for regained response (RR) in this study, defined by an Observer’s Assessment of Alertness/Sedation (OAA/S) score of 4, opens avenues for future research exploring diverse OAA/S thresholds and their impact on predictive accuracy. Further research could delve into the exploration of different drug combinations and the prediction of the time to the loss of consciousness during anesthesia. These prospective avenues aim to advance our understanding of anesthesia dynamics and contribute to the refinement of personalized anesthesia management strategies for enhanced patient outcomes in diverse clinical settings.

## 4. Materials and Methods

### 4.1. Study Group

This was a prospective, observational study with data reanalysis. Twenty-nine patients (American Society of Anesthesiologists (ASA) class II to III) who underwent VATS for elective pulmonary or mediastinal surgery under TIVA were enrolled. The Taipei Veterans General Hospital’s Institutional Review Board (IRB 2023-09-014AC) granted an exemption from the need for written informed consent. The study was carried out in strict adherence to the guidelines approved by the IRB, ensuring that ethical principles and regulations were followed throughout the research process.

We documented demographic information including gender, age, body weight, stature, and ASA classification (Table 1). The patients (11 men and 18 women) were within the age range of 20 to 80 years old. Exclusion criteria for the study included patients who were undergoing emergency surgery and patients with neurological conditions, coagulation disorders, allergies to amide-based local anesthetics, hearing deficiencies, local infections, opioid use, alcohol intake exceeding 20 g daily, or recent intake of psychoactive drugs.

### 4.2. Anesthesia Methods

Patients did not receive any premedication. The induction process began with a Fentanyl bolus dose of 3–5 ug/kg, followed by the use of a target-controlled infusion (TCI) pump for maintaining an effect-site concentration (CeP) of Propofol between 4 and 10 mg/mL during the entire procedure. The TCI pump was adjusted to achieve a target BIS dosage of 40–60. Concomitant effect-site drug concentration (C_e_) and BIS recordings were performed every minute. After confirming the patient’s loss of consciousness, a neuromuscular blocking agent (a single dosage of 0.6–1 mg/kg of Rocuronium) was administered to ease intubation. No regional blockade was performed during or after the induction of anesthesia. The patient was intubated with a double-lumen tube (DLT), followed by the verification of its accurate placement through auscultation and fiber optic bronchoscopy. The TCI pump was adeptly utilized to modulate the Propofol dosage, while intermittent Rocuronium administrations were carefully administered to sustain a singular twitch after a train-of-4 stimulus, thereby ensuring optimal muscle relaxation throughout the anesthesia procedure. This meticulous approach to anesthesia induction underscores a commitment to precise control and patient safety during the entirety of the medical intervention.

Patient monitoring was conducted throughout the whole surgery using electrocardiography, pulse oximetry, and blood pressure measurements. Ventilation was controlled artificially to keep end-tidal CO_2_ at approximately 35–40 mmHg. Body temperature was actively maintained at approximately 35.5 °C to promote a stable physiological environment. Vital signs, patient responses, surgical process, anesthetic drug concentrations, drug administration time, and the drug administered dose were recorded throughout the procedure. This meticulous monitoring strategy not only ensured the safety and stability of the patient but also provided valuable data for real-time assessment and adjustment of anesthesia parameters throughout the entire medical intervention.

Propofol was discontinued after skin closure. Neostigmine (1–2 mg) and Atropine (0.5–1 mg) were administered intravenously to reverse the neuromuscular blockade. The patient’s status was diligently documented using the Observer’s Assessment of Alertness and Sedation (OAA/S) scale [28] every 20 s, with corresponding values and descriptions systematically recorded in Table 2. RR was defined as two consecutive OAA/S scores ≥4. The exact time point of the RR was recorded and the observation was sustained for 10 min after the patient showed RR. Double-lumen tube extubation was performed after sufficient spontaneous ventilation. Subsequently, patients were transitioned to the post-anesthesia care unit (PACU) for a comprehensive monitoring period lasting 2 h. In the PACU, vital signs, incidences of nausea and vomiting, and any signs of bleeding were vigilantly observed to ascertain the patients’ recovery and stability in the immediate post-anesthetic period. Postoperative analgesics were administered either through an intravenous patient-controlled analgesia machine or by employing traditional analgesia methods. The loading dose was administered in the post-anesthesia care unit.

### 4.3. Pharmacokinetic Simulation

The pharmacokinetic profiles for Ce were computed employing the Tivatrainer simulation program (version 8). The Schnider Ce model was used for determining the Propofol target-controlled infusion (TCI) dosage. Additionally, the calculated Ce values for Fentanyl, sustained throughout the anesthesia period, underwent conversion to equivalent Remifentanil concentrations, guided by the established potency ratio of 1:1.2 for Remifentanil to Fentanyl [27,28].

### 4.4. Response Surface Models

RSMs were constructed using the Greco model structure, as shown in the following equation:

$$\mathrm{Effect}=\frac{Emax\times {\left[\frac{CeR}{C50R}+\frac{CeP}{C50P}+\alpha \;\left(\frac{CeR}{C50R}\times \frac{CeP}{C50P}\right)\right]}^{n}}{{\left[\frac{CeR}{C50R}+\frac{CeP}{C50P}+\alpha \;\left(\frac{CeR}{C50R}\times \frac{CeP}{C50P}\right)\right]}^{n}+1}$$

The Greco intravenous Propofol–Remifentanil interaction models, systematically established across a spectrum of concentration pairs, serve as a pivotal tool in the computation of sedation probabilities, specifically focusing on Observer’s Assessment of Alertness and Sedation (OAA/S) scores below. This effect ranges from 0 to 1, indicating the likelihood of no response. Within this intricate modeling framework, E represents the predicted effect at a specific steady-state plasma concentration, with Emax signifying the maximal effect, reflecting a 100% probability of no response, and E0 denoting the baseline effect. C_50_P (Propofol) and C_50_R (Remifentanil) delineate the effect-site concentrations necessary to produce 50% of the maximal effect when Propofol or Remifentanil is administered individually. The parameters N and α represent the steepness of the response surface and the interaction strength between Propofol and Remifentanil, respectively. This comprehensive utilization of Greco models not only facilitates a nuanced understanding of sedation probabilities but also contributes to the refinement of dosing strategies, ultimately enhancing the precision and safety of intravenous anesthesia practices in clinical settings. The parameters of the model are presented in Table 3.

For each patient, the predicted probabilities of regained response (RR) were derived by transforming the probabilities of loss of response (LOR), ranging from 0% to 100%, through the calculation of 1 minus the probability of LOR. We calculated the model predictions for OAA/S ≥ 4 at which each RSM predicted a 50% probability of response and compared the predicted RR times with the observed time when patients with lethargy responded to their names during emergence. To assess the model’s predictive accuracy, we constructed a receiver operating characteristic (ROC) curve by closely examining the relationship between the predicted probabilities and the actual observed values.

### 4.5. Logistic Regression

Data and statistical analyses in this study were conducted using IBM SPSS Statistics 20. The predictive model employed concentrations of Propofol and Remifentanil in blood at different time points to calculate the probability of the return of responsiveness (RR). Descriptive statistics, including mean values, standard deviations, and 95% confidence intervals, were computed for metric variables, while categorical variables underwent assessment for significant associations using the chi-square test. The process of selecting variables was conducted using a forward selection method, adhering to a predetermined *p*-value threshold of 0.05 for determining inclusion. This approach automatically adds or removes variables contingent upon their significance and contribution to the model. This method automatically selects variables for inclusion or exclusion by calculating their respective contributions to the model. To evaluate the model’s predictive accuracy, a receiver operating characteristic (ROC) curve was constructed by scrutinizing the relationship between predicted probabilities and actual observed values.

### 4.6. Comparison

Finally, the ROC curves of the LR model and the RSM were compared to discern any divergences in their predictive capabilities. This robust analytical framework, combining sophisticated statistical methods and model comparisons, forms a comprehensive strategy for refining and validating the predictive accuracy of the concentration-based RR model in anesthesia research.

## 5. Conclusions

The RSM is highly effective for modeling and optimizing complex processes. Its ability to handle multiple input variables and their non-linear relationships and interactions results in exceptional accuracy. In contrast, logistic regression, though effective for binary classification problems, has limitations compared to the RSM. It primarily addresses linear relationships and predicts the probability of binary outcomes, lacking the RSM’s capacity to model complex, non-linear interactions among multiple variables.

In our study, we limited our analysis to the effects of Remifentanil and Propofol in predicting patient emergence. By simplifying the parameters, the LR model was effective, and it can be applied to patients undergoing VATS or other procedures with similar modalities. While the RSM exhibits greater sophistication, achieving comparable accuracy to the LR model, the latter proves more accessible. However, for more detailed or numerous parameters, logistic regression would be less suitable. The models can be employed in computer simulations to devise customized optimal dosing regimens. The LR model emerges as a simpler yet effective tool for predicting arousal in patients undergoing VATS under TIVA with Remifentanil and Propofol.

## Figures and Tables

**Table 1:** Patient demographic profile.

Parameter	Value
Age, years	54.6 ± 11.6
No. of males	11
No. of females	18
Weight, kg	60.6 ± 12.2
Height, kg	159.5 ± 8.8
Body mass index, kg/m^2^	23.8 ± 64.4
No. ASA physical status I	0
No. ASA physical status II	20
No. ASA physical status III	9
Operation time (minutes)	198 ± 43

ASA = American Society of Anesthesiologists.

**Table 2:** Observer’s Assessment of Alert/Sedation (OAA/S).

Value	Description
5	Response readily to a name spoken in a normal tone.
4	Lethargic response to name spoken in normal tone.
3	Responds only after the patient’s name is called loudly and/or repeatedly for the individual to open their eyes.
2	Responds only after moderate prodding or shaking.
1	Does not respond to moderate prodding or shaking.
0	Does not respond to deep stimulus.

Regained response (RR) was defined as two consecutive OAA/S scores ≥4.

**Table 3:** Propofol–Remifentanil interaction model parameters for responses recorded in volunteers for OAA/S < 4.

Model Parameters	C_50_P (mg/mL)	C_50_R (ng/mL)	N	Alpha
OAA/S < 4	1.8	12.5	3.8	5.1

OAA/S = Observer’s assessment of Alertness and Sedation scale; C_50_P and C_50_R = Propofol and Remifentanil concentrations associated with a 50% probability of effect; N and alpha = model parameters representing the steepness of the dose–response relationship and the interaction between Sevoflurane and Remifentanil.

## Data Availability

Data are contained within the article.
